# Tuberculous Miliary Disease Following Intravesical Bacillus Calmette-Guérin Therapy for Urothelial Carcinoma of the Bladder: A Case Report

**DOI:** 10.7759/cureus.109252

**Published:** 2026-05-20

**Authors:** Achraf Benamou, Anouar El Moudane, Ali Barki

**Affiliations:** 1 Urology, Mohammed VI University Hospital, Oujda, MAR

**Keywords:** bladder cancer, infection, intravesical bcg therapy, miliary tuberculosis, systemic complications

## Abstract

Intravesical instillation of Bacillus Calmette-Guérin (BCG) is the standard adjuvant treatment for non-muscle-invasive bladder cancer. Although highly effective, BCG therapy may be associated with rare but potentially severe systemic complications. We report the case of a 52-year-old man treated with intravesical BCG for carcinoma in situ of the bladder who subsequently developed miliary tuberculosis. This case shows that general complications of intravesical BCG therapy are becoming increasingly frequent and should be used with caution while respecting international recommendations.

## Introduction

Since its first clinical use by Morales et al. in 1976, intravesical Bacillus Calmette-Guérin (BCG) immunotherapy has become the treatment of choice for high-risk non-muscle-invasive bladder cancer, particularly carcinoma in situ and recurrent or multifocal tumors [1]. The antitumor efficacy of BCG is mediated through the induction of a complex local immune response involving both innate and adaptive immunity within the bladder mucosa [2].

Despite its proven efficacy, BCG therapy is associated with adverse effects in up to 60-90% of patients, most of which are mild and localized to the lower urinary tract [3]. Systemic complications are uncommon, occurring in fewer than 1% of cases, but may be severe and life-threatening, including BCG sepsis, granulomatous hepatitis, and miliary tuberculosis [4].

## Case presentation

A 52-year-old man undergoing treatment for non-muscle-invasive bladder cancer of the carcinoma in situ type received weekly intravesical instillations of BCG. Following the second instillation, he developed marked intolerance characterized by pelvic discomfort, persistent high-grade fever (39°C), and severe asthenia. Laboratory investigations revealed an elevated C-reactive protein level of 156 mg/L and leukocytosis of 14,300/mm³.

**Figure 1:**
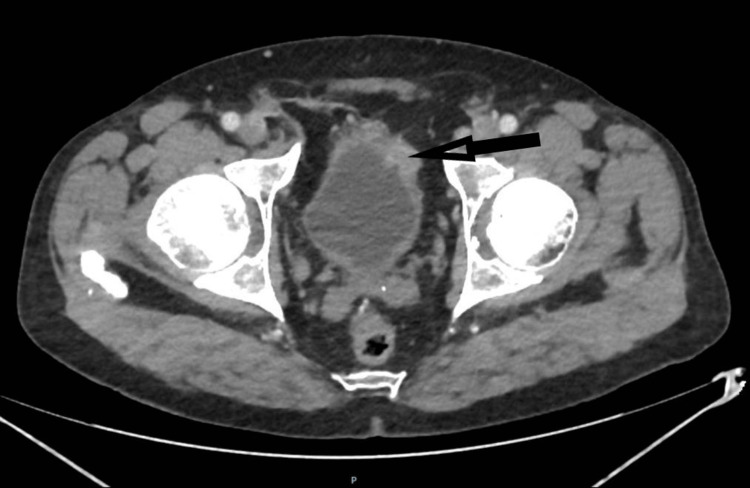
Computed tomography scan image showing a tumor on the anterior wall of the bladder

**Figure 2:**
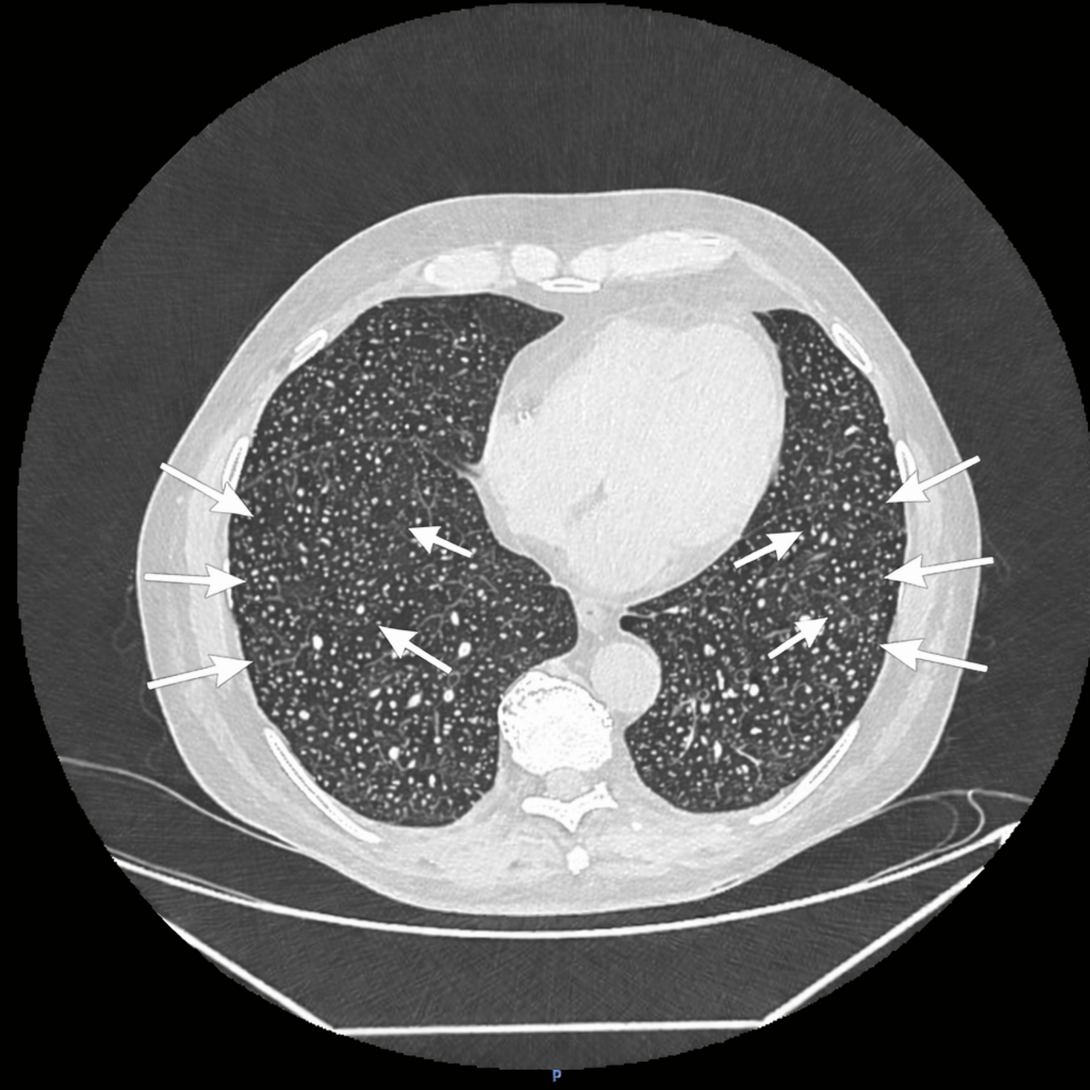
Miliary tuberculosis on the chest computed tomography scan

A urographic computed tomography (CT) scan revealed a developing lesion on the anterior wall of the bladder (Figure 1). Empirical broad-spectrum antibiotic therapy was initiated; however, the patient's clinical condition failed to improve. Given the persistence of fever, a chest CT scan was performed and revealed a diffuse micronodular pattern consistent with miliary tuberculosis (Figure 2). Acid-fast bacilli were subsequently identified in sputum samples, confirming mycobacterial infection.

The patient was transferred to the pulmonology department, placed in respiratory isolation, and initiated on standard quadruple antituberculous therapy consisting of isoniazid, rifampicin, ethambutol, and pyrazinamide. Clinical and biological parameters improved progressively, with complete resolution of infectious signs after two months of treatment.

## Discussion

The BCG strain used for intravesical immunotherapy is a live attenuated form of *Mycobacterium bovis* and represents one of the most effective adjuvant treatments for high-risk non-muscle-invasive bladder cancer. Its antitumor efficacy is mediated through a complex immune cascade involving urothelial internalization of BCG, activation of antigen-presenting cells, and subsequent recruitment of innate and adaptive immune responses, ultimately leading to tumor cell destruction [5]. Despite these benefits, the use of live mycobacteria carries an inherent risk of infectious complications.

Systemic dissemination of BCG is rare but well documented. Several mechanisms have been proposed to explain hematogenous spread, including traumatic catheterization, early instillation following transurethral resection, active cystitis, or gross hematuria, all of which may facilitate mucosal breach and vascular invasion by viable bacilli [6]. In the present case, the temporal relationship between BCG instillation and symptom onset strongly supports a causal association.

Miliary tuberculosis following intravesical BCG therapy is an exceptionally uncommon manifestation, accounting for a very small proportion of reported systemic complications. Its clinical presentation is often insidious and nonspecific, typically characterized by persistent fever, asthenia, weight loss, and occasionally respiratory symptoms such as dry cough or dyspnea [7]. This lack of specificity frequently leads to diagnostic delay, as symptoms may initially be attributed to common post-BCG inflammatory reactions or bacterial infections.

Radiological findings play a pivotal role in raising diagnostic suspicion. Chest CT usually reveals diffuse bilateral micronodular infiltrates consistent with a miliary pattern, as observed in our patient. Definitive diagnosis relies on microbiological confirmation through the identification of acid-fast bacilli in respiratory samples or molecular techniques such as polymerase chain reaction [8]. Blood cultures are often negative, further complicating the diagnostic process.

The cornerstone of management is the immediate discontinuation of BCG therapy and prompt initiation of antituberculous treatment. Although *Mycobacterium bovis *is intrinsically resistant to pyrazinamide, standard quadruple therapy is frequently initiated empirically until species identification is confirmed, after which treatment may be adjusted accordingly [9]. In severe cases, including disseminated or life-threatening forms, adjunctive corticosteroid therapy has been shown to improve outcomes by mitigating the inflammatory response [10].

Preventive measures remain essential to reduce the risk of systemic BCG complications. Current recommendations emphasize delaying BCG instillation in the presence of active urinary tract infection, macroscopic hematuria, or traumatic catheterization, as well as strict adherence to aseptic techniques during administration [11,12]. Careful patient selection and close post-instillation monitoring are crucial, particularly during the early treatment cycles.

This case highlights the need for heightened clinical awareness of rare systemic complications associated with intravesical BCG therapy. Early recognition and multidisciplinary management involving urologists, infectious disease specialists, and pulmonologists are key to ensuring favorable outcomes in these potentially life-threatening situations.

## Conclusions

Miliary tuberculosis is a rare but potentially fatal systemic complication of intravesical BCG therapy for non-muscle-invasive bladder cancer. Although intravesical BCG is generally safe and remains a cornerstone of bladder cancer management, clinicians must remain vigilant for the possibility of disseminated infection, particularly in patients presenting with persistent, unexplained fever, constitutional symptoms, or signs of systemic inflammation following BCG instillation.

This case aims to raise awareness within the urological community that systemic complications of BCG therapy, while uncommon, may be encountered with increasing frequency. Importantly, many of these adverse events are preventable through strict adherence to established international guidelines for patient selection, timing, and administration of intravesical BCG.
